# Does the Level of Confidence Exhibited by Dental Students Predict the Outcome of Complete Denture Therapy?

**DOI:** 10.1155/2020/9752925

**Published:** 2020-07-17

**Authors:** Indika P. Thilakumara, Kaumudi U. Prathibhani, Sumudu G. K. Rasnayaka, Sachith P. Abeysundara, Rasika M. Jayasinghe

**Affiliations:** ^1^Faculty of Dental Sciences, University of Peradeniya, Peradeniya, Sri Lanka; ^2^Teaching Hospital, Anuradhapura, Sri Lanka; ^3^Faculty of Science, University of Peradeniya, Peradeniya, Sri Lanka

## Abstract

**Materials and Methods:**

Fifty-seven final-year dental students in the year 2019 and the first edentulous patient managed by them were recruited for the study. A fourteen-item questionnaire was given to the students to assess their level of confidence just before commencement of the treatment. Questionnaire for the patients was used to assess their satisfaction both with the operator and the dentures. Clinical outcome of dentures was assessed using ten criteria.

**Results:**

The average confidence level of the students was found to be 2.17 in a 4-point scale. A two-sample test indicated that there is a significant difference in the overall level of confidence between female and male students (*p* value = 0.004). Moreover, a nonparametric correlation analysis revealed that there is no significant correlation between the overall level of confidence of each student and patient satisfaction regarding student performance, level of satisfaction of patients regarding dentures, and the quality of dentures as assessed by the clinicians (*p* value = 0.545, 0.877, and 0.801, respectively).

**Conclusions:**

Students' level of confidence in carrying out complete denture procedures is satisfactory. Male students exhibited a better overall level of confidence than female students. However, the level of confidence exhibited by the dental students does not predict patient satisfaction or clinical outcome.

## 1. Introduction

Developing competence in technical and clinical skills and building up self-confidence are considered important attributes of the graduate profile of many dental schools since dentistry is fundamentally skill-based health science [1]. Chambers defined clinical competency as the ability of the students to carry out clinical procedures independently which builds the bridge between education and practice [2]. Clinical confidence is the self-perceived ability to deal with clinical scenarios. However, it does not necessarily correlate with competency; it is nevertheless a pre-requisite for students to be able to fully participate in clinical activities [3]. Improvement of self-confidence related to clinical activities is considered as an essential element in teaching dentistry [4].

Various authors have discussed about the relationship of stress and anxiety in relation to enhancement of clinical skills in dentistry. Dental professionals might experience occupational stress from their interaction with staff and patients, concerns in patient management, time pressure, and paper work, as well as concerns about defective equipment [5]. Eliminating stress totally in a dental education program is hard. Furthermore, dental undergraduates have to achieve appropriate levels of knowledge and skill as well as to improve good attitude towards patients they manage within a short period of time to build up a successful dental professional career [6]. Therefore, considering all these aspects, the curriculum should provide a solid foundation to build the confidence of undergraduate students, in preparation for their professional life [7].

In addition, patient perception seems to be fundamental when assessing the success of any therapy. Thus, patients' satisfaction with their complete dentures may be considered the ultimate goal of complete denture therapy [8]. Factors affecting patient satisfaction in a dental clinic have been studied with the aim of improving the quality of the treatment. Gurdal et al. showed that personal interactions between the patient and the clinician play a major role in satisfaction, irrespective of the cultural and ethnic background of the clinician [9]. Previous studies have also shown that the experience of the clinician, the patient's opinion of the clinician, and the communication skills of the clinician influence patient satisfaction [10]. However, only few studies have explored patient satisfaction related to treatments carried out by the dental students [11, 12].

As provision of replacement options such as implant-supported overdentures is limited due to economic constraints, conventional complete denture therapy continues to remain a viable treatment option for the routine edentulous patient. Therefore, teaching conventional complete denture therapy can be expected to remain in dental curricula for some time. In the Faculty of Dental Sciences, University of Peradeniya, where this study had been carried out, edentulous patients are managed with conventional complete dentures by the final-year dental students under the supervision of a teaching consultant.

It can be expected that improving student confidence prior to patient treatment together with sound theory and clinical skill might lead to an acceptable level of patient satisfaction at the end of the treatment. Studying the relationship between the level of confidence of students and patient satisfaction with complete dentures could provide incentive for future curriculum revision and improve the service towards the patients.

The aim of this study was to investigate the relationship between the level of confidence of dental students, the satisfaction level of patients, and the actual clinical outcome of complete dentures. Our specific objectives were to assess the relationship between the

- Level of confidence of dental students and patients' satisfaction with complete dentures
- Level of confidence of dental students and patients' satisfaction with student performance
- Level of confidence of dental students and the clinical outcome of complete dentures provided by them
- Clinical outcome of complete dentures and patients' satisfaction with them

## 2. Materials and Methods

This study was approved by the Ethical Review Committee of the Faculty of Dental Sciences, University of Peradeniya (ERC/FDS/UOP/I/2018/15).

### 2.1. Study Design, Setting, and Selection of Study Participants

A descriptive cross-sectional study was conducted which included fifty-seven final-year dental students of age 24–26 yrs in the year 2018 at the Faculty of Dental Sciences, University of Peradeniya, and the first edentulous patient managed by them. The students had already had lectures and clinical and laboratory demonstrations on the management of the edentulous patient with complete dentures.

They were allocated to the study considering the inclusion and exclusion criteria, and informed written consent was obtained prior to the study. The study was carried out conforming the STROBE Guidelines and ethical standards of the 1964 Helsinki Declaration.

### 2.2. Inclusion Criteria

- Final-year dental students managing their first edentulous patient
- Edentulous patients allocated for the above students

### 2.3. Exclusion Criteria

- Final-year students who had already commenced the treatment for patients requiring complete dentures

### 2.4. Collection of Data

This study was carried out in three stages.

#### 2.4.1. Stage 1: Assessing the Level of Confidence of the Students

All participating students were given a pretested and validated questionnaire (Table 1: student questionnaire) prior to commencement of the treatment procedure. The self-administered questionnaire consisted of fourteen statements. The students used a Likert scoring system from 1–4 to indicate their level of confidence as follows: 1 very good, 2 good, 3 satisfactory, and 4 poor. An overall mean score was calculated to rank the responses.

#### 2.4.2. Stage 2: Assessing the Level of Satisfaction of the Patients

All participating patients were given a pretested and validated questionnaire (Table 1: patient questionnaire) to determine their level of satisfaction. Other than the statements regarding their satisfaction with the student, dentures, and the institution, it also included questions regarding gender and age. This questionnaire was provided in the patient's preferred language (Sinhalese, Tamil, and English) as an interviewer-administered questionnaire. Statements 1–12 were administered at the denture insertion stage, and statements 13–15 were given at the first postinsertion review stage. Patients rated their satisfaction in a four-point Likert scale (1-strongly agree, 2-agree, 3-disagree, and 4-strongly disagree).

#### 2.4.3. Stage 3: Assessment of the Clinical Outcome

A checklist with 10 criteria was used at the postinsertion review stage to assess the clinical outcome (Table 1: clinical outcome). Assessment was carried out by two specialists in the field. Each criterion was rated in a five-point Likert scale, 1 being very satisfactory to 5 being very unsatisfactory.

The study involved the calculation of the correlation coefficient between the level of confidence of students' and the patients' satisfaction. Hence, hypothesis testing was involved to compare the two groups. Hulley et al. (2013) and Suresh and Chandrasekara (2012) discussed the sample size calculations in clinical research studies [13, 14]. Based on that, the required sample size was determined based on the power of the test (0.90), the significance level (0.05), and the expected strength of the correlation coefficient (0.50, a moderate positive relationship). Hence, it was decided that at least responses from 38 pairs were to be collected to achieve the objectives of the study. Therefore, a total of fifty-seven student-patient pairs were considered as participants of the study. Three of the patients did not complete the postinsertion review and were therefore excluded from the study. Both questionnaires were administered on a voluntary and anonymous basis.

### 2.5. Data Analysis

Data were analyzed using Minitab (v18.0) statistical software. As the scores for more than one response were added, the mean of the total score was considered. Hence, parametric correlation analysis was done to find the significance of the relationship. Moreover, ANOVA and association tests, as well as regression analysis, were used to answer the secondary objectives. When the distributional assumptions on data were not satisfied, the equivalent nonparametric tests such as Mann–Whitney *U* test, Kruskal–Wallis, and Spearman correlation were used for the analysis. Chi-square test was used to test the association of two Likert-type responses. Level of significance was considered as *p* < 0.05.

## 3. Results

Among fifty-seven students, 56.1% (32) were female respondents. Out of fifty-four patients, 63.0% (34) were females.

### 3.1. Level of Confidence of the Students

**Figure 1:**
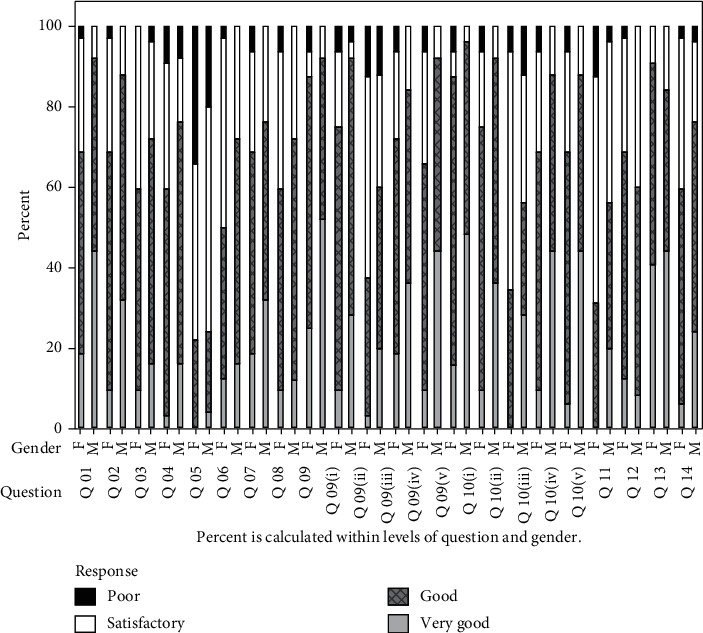
Level of confidence among dental students of the two genders to carry out the complete denture procedure.

Level of confidence of the students pertaining to the prosthodontic management aspects was assessed for each of the 14 statements (Table 1: student questionnaire) separately (see Figure 1). The results revealed that there is a significant difference between the two gender groups (at 0.05 level) in the median levels of confidence in relation to competence in gathering and interpreting information obtained from the patient, carrying out, reporting, and interpreting examination findings, knowledge to carry out treatment steps, practical skills to carry out treatment steps, and carrying out treatment procedures on their own without supervision.

Considering all fourteen statements, the average confidence level of the students was found to be 2.17 in a 4-point scale. Furthermore, a two-sample test indicated that there is a significant difference in the average confidence level for the female and male student groups where male students exhibited a better overall level of confidence than female students (*p* value = 0.004) (Table 2).

Statements 1–5 and 8 (Table 1: student questionnaire) encompassed student's confidence in gathering information by history taking, examination, and carrying out investigations as well as in treatment planning. Therefore, the analysis is performed for these statements collectively and is reported as the median of the confidence scores of the above statements for each individual. A 95% confidence interval is then calculated. The results indicate that the median level of confidence falls within 2.0 and 2.5 suggesting that the student's level of confidence in carrying out these procedures is good.

When considering the treatment steps separately, there was a significant difference in the confidence level between the two genders for competence in gathering and interpreting information obtained from the patient (*p* value 0.011) and interpreting examination findings (*p* value 0.016), knowledge to carry out treatment steps; primary and secondary impression, and denture delivery (*p* values 0.049, 0.029, <0.001, respectively), and practical skills to carry out treatment steps; primary and secondary impression, trial denture, and denture delivery (*p* values 0.007, 0.008, 0.003, and 0.001, respectively). There was also a significant difference between the genders with regard to confidence in carrying out treatment procedures on their own without supervision (*p* value 0.018).

When considering students' level of confidence regarding their knowledge to carry out each treatment step and in application of practical skills during patient management, a two-sample test indicates that there is a significant difference in the confidence level for both statements between the female and male student groups (*p* values 0.008 and 0.003, respectively). The confidence level associated with the knowledge component was greater compared with the skill component of the same procedure.

A 95% confidence interval is calculated for the median level of confidence of students in communication with the patient, and the obtained interval is 2.0–2.5 indicating that students' level of confidence with regard to communication with patients is good.

### 3.2. Level of Satisfaction of Patients and Its Correlation with the Level of Confidence of Students

**Figure 2:**
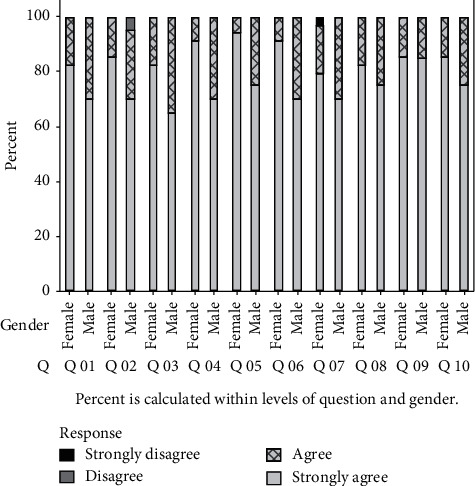
Comparison between the level of satisfaction between male and female patients regarding students' performance.

The level of satisfaction of patients regarding students' performance was assessed using statements 1–10 (Table 1: patient questionnaire) (see Figure 2). The responses for the 10 statements are averaged among the 54 patients, and the average response is found to be 1.2 in a 4-point scale. Hence, it can be concluded that the patients have a high level of satisfaction with the students' performance.

A statistical analysis was conducted to see whether there is a significant difference between the two gender groups for each statement. Results revealed that a statistically significant difference (at 0.05 level) in the median levels of satisfaction of the two gender groups exists when considering students' examination of them without causing any discomfort, respect to the them during the clinical procedures at the chair side, and students' level of understanding about clinical problems.

Statements 1–3 in the patient questionnaire aim to assess patient's satisfaction regarding the students' communication skill. These statements were therefore assessed collectively. The 95% confidence interval (1.12–1.30) for the average satisfaction regarding these statements indicated that the patients are very satisfied with the communication skills of the students.

**Figure 3:**
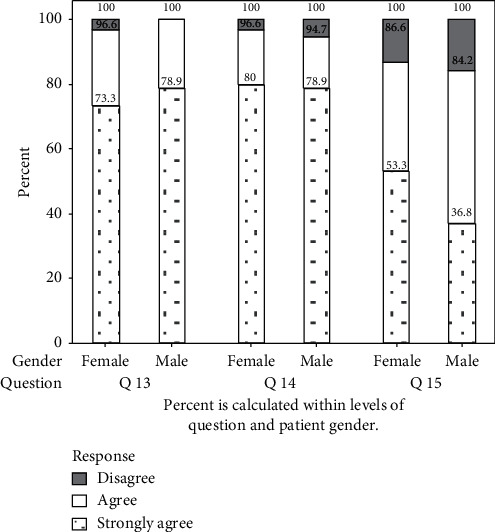
Overall level of satisfaction for statements 13–15.

Furthermore, when statements 13–15 with regard to patient satisfaction in the final denture were analyzed together and reported as the sum of the satisfaction scores of the above statements for each individual, levels were defined as follows: if the sum is ≤3: strongly agree, 4–6: agree, and ≥7: disagree, and the results indicated that the overall satisfaction level falls within 4.0 and 4.5 (Figure 3).

A statistical analysis revealed that all the patients “strongly agree” on statement 11, satisfaction regarding quality and standard of treatment provided for them, and statement 12 recommendation of institute to others.

To assess the correlation between the confidence of students to communicate effectively with patients and the patients' overall satisfaction regarding communication skills of students, a nonparametric correlation analysis was carried out. It revealed that there is no significant correlation (*p* value = 0.639).

Similarly, a correlation analysis found that there is no significant correlation between the overall level of confidence for each student and overall level of satisfaction of each patient regarding the treatment by students (*p* value = 0.545). Moreover, it is found that there is no significant correlation between the overall level of confidence for each student and overall level of satisfaction regarding dentures of each patient (*p* value = 0.877).

### 3.3. Clinical Outcome and Its Correlation with the Level of Confidence of Students and the Level of Satisfaction of Patients

**Figure 4:**
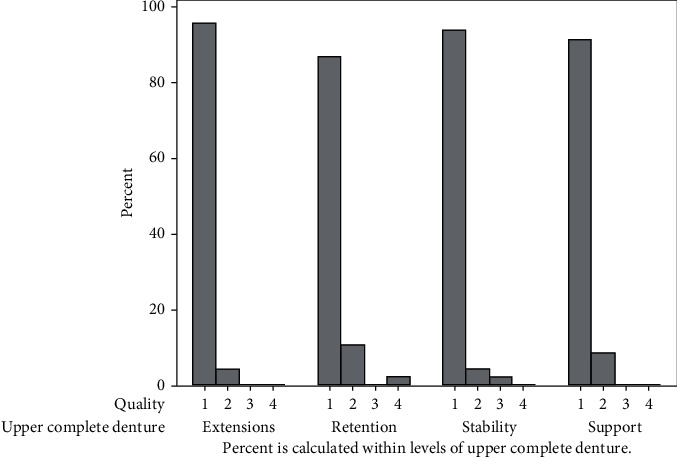
Level of satisfaction with the upper complete denture in relation to stability, support, and extensions.

**Figure 5:**
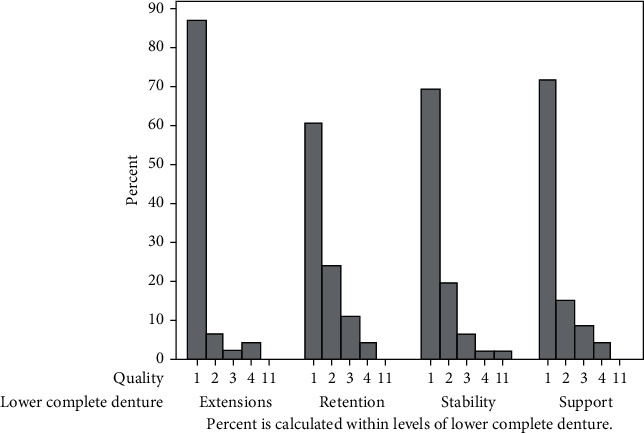
Level of satisfaction with the lower complete denture in relation to retention, stability, support, and extensions.

When the maxillary dentures were assessed for their quality, it was revealed that more than eighty percent of them had very satisfactory retention, stability, support, and border extensions (Figure 4) compared with sixty percent in mandibular dentures (Figure 5).

When the relationship between the level of confidence of students and the quality of the maxillary denture was analyzed, we were unable to identify any significant association (*p* value = 0.801). Similarly, there was no association between the level of confidence of students and the quality of the mandibular denture (*p* value = 0.539).

A significant weak positive correlation (*r* = 0.316, *p* value = 0.033) between the qualities of retention, stability, support, and extension of maxillary dentures and patients' perception for fit of the maxillary dentures was observed. On the contrary, no such correlation was seen for mandibular dentures (*p* value = 0.350).

According to the assessment of the outcome by the clinicians, ninety-eight percent of dentures had very satisfactory shade and mold. When we assessed the association between the student's level of confidence with selection of appropriate shade and mold, the results failed to show any significant association between the level of confidence and the clinical outcome regarding complete denture aesthetics (*p* value = 0.219).

Finish of both maxillary and mandibular dentures was very satisfactory. Balanced occlusion had been achieved to a very satisfactory level in 76% of the dentures. 52.1% of the dentures showed a very satisfactory level of balanced articulation. 82.6% of the dentures had the recommended freeway space of 2–4 mm.

Moreover, there was no statistically significant correlation between the balanced occlusion, balanced articulation, and availability of adequate freeway space of maxillary and mandibular dentures and patients' perception in relation to the ability to perform oral function using the dentures (*p* value = 0.676).

## 4. Discussion

All the students in this study were taught the theoretical aspects of complete denture procedures prior to their clinical appointment. Similarly, clinical demonstrations for each stage of complete denture fabrication have also been carried out for them by a supervising consultant. They have also practiced laboratory procedures of complete denture fabrication on standard casts and have gained clinical skill to a certain extent by management of two partially dentate patients with removable dentures in the previous year. In the final year, they are required to provide conventional complete dentures for two patients under the supervision of the academic staff members. Our study was carried out with fifty-seven final-year dental students prior to commencement of their first complete denture case.

Thus, we shall expect all the students to have the same knowledge and the same clinical exposure of complete denture therapy at the baseline and shall expect them to exhibit similar competence level. Our results indicated that the overall confidence level of the students is good. This is compatible with the results of the previous studies where the authors have demonstrated that students reported highest confidence in simple procedures [15]. However, it is contrary to studies that show that students reported highest confidence in procedures in which they had the most clinical experience [13]. Murray et al. who reported insufficient clinical exposure as one of the limits to develop confidence in performing clinical practices supported the above finding [14]. It has also been shown that the level of confidence increases as the students progress in their clinical training [16]. A study assessing the need of information on dental implants has identified that all respondent undergraduates mentioned that they were not provided with enough details about implant treatment procedures during their BDS program. Therefore, they felt that more need of information on the topic is required for them to develop competency [17]. Similarly, though we expect all to improve knowledge on management with complete denture, it may not be successful all the time. Therefore, more information would provide a sufficient platform for more confidence in carrying out the procedure.

Our results indicated that there was a significant difference in the overall confidence level for female and male student groups. Male students exhibited a better overall level of confidence than female students. Previous studies have shown male students are more confident with the procedures related to problem solving, and female students are more confident with caries detection, rubber dam placement, preventive resin and simple posterior restoration, and retreatment of failed RCT [3].

In all the five clinical stages, male students showed better level of confidence both in knowledge and skills compared with female students. In addition, confidence level associated with the knowledge component was greater compared with the skill component of the same procedure. This is expected as the confidence related to skills usually builds up with long-term practice [16]. Students' level of confidence would get improved with increased clinical exposure. However, both gender groups demonstrated low confidence of knowledge to carry out the treatment step of registering of maxillary and mandibular relations and low confidence of practical skills to carry out the same. This highlights the necessity to improve both the knowledge and the clinical skills in relation to registration of maxillary and mandibular relations in complete denture fabrication.

Self-perceived confidence level does not necessarily mean that one is competent in the particular domain being tested. The quality of the denture and patient satisfaction with the dentures are the ultimate outcomes of complete denture therapy, hence can be considered as a means of assessing the competence of the operator. Although our results revealed that the overall confidence level of the students and the overall satisfaction of the patients were good, there was no correlation between the two. Students' perceptions of confidence levels are subjective, and there can be a possible individual variation in the interpretation of the questionnaire and also when rating it in Likert's scale. There are contrasting views as to whether having high levels of confidence can correlate with successful performance [18, 19]. There have been reports on students being overconfident and putting their patients at risk when carrying out treatment procedures [18]. Previous studies have shown that the confidence level of the students could be affected by the factors such as comments of the tutor and the outcome of the treatment [19]. In this study, the confidence level was assessed prior to commencement of the treatment; thus, the possibility of such influence is remote.

In addition, the assessment carried out by the examiners using ten criteria denoted that the dentures were of good quality. However, when the level of confidence of students was analyzed with the quality of maxillary and mandibular dentures in terms of retention, stability, support, and extension, we were unable to identify any significant association. Thus, it is obvious that the level of confidence exhibited by dental students does not predict either the clinical outcome of the dentures or patient satisfaction with them. There could be two explanations for this finding. Students carry out treatment procedures under supervision; hence, they would have made it a point to see that the end result is good. Secondly, the students may underestimate themselves. Thus, they need to be motivated in this regard to boost their level of confidence.

There are multiple research studies available in the literature related to perceived stress, anxiety, and information need.

A recent study was carried out in New Zealand using fourth-year undergraduate dental students who were randomly assigned into two intervention groups: cognitive reappraisal intervention/experimental (EXP) and control intervention (CON) groups and identified that the EXP group showed a small-to-medium decrease in the perceived level of stress in comparison with the CON group. However, the results were not statistically significant [20].

A study has highlighted that prior preparation of the students to cope with stress resulting from handling and management of the patients is of high importance. The researchers have recommended that such training programs should be implemented before the commencement of practical clinical sessions. They further state that the acquired knowledge will be useful in future professional career [21]. Similarly, another study has revealed that although the dental undergraduates were with positive attitudes on biomedical waste management, lack of knowledge and practice would act as barriers. Therefore, the authors have recommended that more training programs have to be implemented in order to improve those aspects of learning, further highlighting the importance on provision of higher level of knowledge and skills in the dental program [22].

This study also revealed that there is no statistically significant difference between the clinical outcome, quality of the denture in terms of patient satisfaction on the fit of the mandibular denture, esthetics, and the ability to perform oral functions when examined at the postinsertion review which was two weeks after denture insertion. However, a weak correlation was seen with regard to qualities such as retention, stability, support, and the extensions of the maxillary denture and patient satisfaction on the fit of the maxillary denture. Generally, our results do not support a statistically significant relationship between the clinical outcome/denture quality and patient satisfaction even during the initial postinsertion stage. There are both positive and negative relations reported in the literature in this regard. A positive relationship between denture quality and patient satisfaction has been shown after three months after insertion [23, 24]. During a subsequent study, one of the authors has concluded that the effect is not seen two years after insertion [25]. According to Van Waas, satisfaction with the complete denture therapy is individually determined [23]. Even though previous studies have explored several factors related to therapy, patient, and operator, there seems to be no consensus as to the predictors of satisfaction with complete denture therapy. Furthermore, the present report tested conventional technique. However, new CAD/CAM systems registered are constantly increasing for use in dentistry. This technology allows a completely digital workflow from impression to the final framework with acceptable clinical reliability [26] and excellent patients' feedback [27].

### 4.1. Limitations

Regarding the patient perception, there might be an element of bias considering the cultural input affecting countries like Sri Lanka. Patients may lie or be reluctant to express their genuine idea about the prosthesis or the student considering the cost-effective service provided by the institute. They might also think that expressing their genuine idea will lead to poor grades for the student. This can be considered as a limitation of this study. Furthermore, though we expected everyone to develop similar competency on the complete denture clinical procedure, the knowledge provided in the BDS program may not be sufficient for some students to develop competency at an appropriate level. It would be more beneficial if the view of academic staff on each student's confidence on clinical steps was considered.

Use of conventional technique in the present study could also be considered as a limitation. It would also be interesting and beneficial if the students' level of confidence for these emerging technologies could be assessed in the future.

Overall, the authors have observed that the course considered met the program learning outcomes in terms of communication skills, confidence, empathy, and professionalism of the students in addition to traditionally considered knowledge and skill components.

## 5. Conclusion

Students' level of confidence in carrying out complete denture procedures is satisfactory. Male students exhibited a better overall level of confidence than female students.

Patients were very satisfied with the communication skills of the students. However, there was no significant correlation between the overall level of confidence for each student and overall level of satisfaction regarding dentures of each patient.

There was a significant weak positive correlation between the denture qualities of retention, stability, support, and extension of maxillary dentures and patients' perception for the fit of the maxillary dentures. However, there was no statistically significant correlation between the balanced occlusion, balanced articulation, and availability of adequate freeway space of maxillary and mandibular dentures and patients' perception regarding the ability to perform oral function using the dentures.

### 5.1. Observations and Recommendations

It is highly important that the undergraduate dental program contains learning outcomes in terms of communication skills, confidence, empathy, and professionalism of the students in addition to traditionally considered knowledge and skill components. These skills achieved by the students are useful when they manage the patients during the final year.

## Figures and Tables

**Table 1:** Questionnaire showing the Likert scale for the level of confidence of students, level of satisfaction of patients, and clinical outcome of dentures.

	Item	Likert scale

Student questionnaire: confidence skills	1. Competence in gathering and interpreting information obtained from the patient	1 = very good to 4 = poor
	2. Carry out, report, and interpret examination findings	
	3. Appropriate investigations required for the identified problems	
	4. Come up with a correct order of clinical problems to be addressed prior to provision of the dentures	
	5. Identifying other treatment options suitable for the patients apart from conventional complete dentures	
	6. Implementing a customized treatment plan	
	7. Understanding about the principles behind carrying out treatment techniques/methods	
	8. Ability to identify special clinical indications in your patients	
	9. Knowledge to carry out treatment steps	
	10. Practical skills to carry out treatment steps	
	11. Able to carry out treatment procedures on your own without supervision from your supervisor	
	12. Identify your mistakes during clinical time on your own and the ability to rectify them	
	13. Communicate effectively with your patients at the chair side	
	14. Manage time while carrying out different treatment steps	

Patient questionnaire: satisfaction level	1. Explained about the whole treatment procedure	1 = strongly agree to 4 = strongly disagree
	2. Provided a fair opportunity to express concerns and clarify your doubts about treatment steps	
	3. Gathered information from you in a methodical manner with ease	
	4. Examined in an acceptable way without causing any discomfort	
	5. Respected you during the clinical procedures at the chair side	
	6. Had a good understanding about your clinical problems when carrying out the treatment	
	7. Communicated and educated you about the treatment in a commendable manner	
	8. Provided adequate clear postdenture delivery instructions regarding maintenance of the denture and its hygiene	
	9. Confident about the dental student's respect for confidentiality	
	10. Had a good ability on time management during the appointments	
	11. Satisfied with the quality and standard of the treatment provided to you	
	12. Recommend this institutional care to the others for a similar type of treatment	
	13. Satisfied with the fit of the denture provided to you	
	14. Satisfied about the esthetics of the denture	
	15. Satisfied about the capability to perform activities like biting and speech with your denture	

Clinical outcome criteria	1. Retention	1 = very satisfactory to 5 = very unsatisfactory
	2. Stability	
	3. Support	
	4. Extensions	
	5. Shade of artificial teeth	
	6. Mold of artificial teeth	
	7. Finish	
	8. Balanced occlusion	
	9. Balanced articulation	
	10. Freeway space	

**Table 2:** *p* values showing the level of students' confidence on management of patients with complete dentures at different steps.

Different steps in complete denture treatment	*p* value
Competence in gathering and interpreting information obtained from the patient	0.011
Carry out, report, and interpret examination findings	0.016
Knowledge to carry out the step of primary impression for complete dentures	0.049
Knowledge to carry out the step of secondary impression for complete dentures	0.029
Knowledge to carry out the step of denture delivery for complete dentures	<0.001
Practical skills to carry out the step of primary impression for complete dentures	0.007
Practical skills to carry out the step of secondary impression for complete dentures	0.008
Practical skills to carry out the step of denture trial for complete dentures	0.003
Practical skills to carry out the step of denture delivery for complete dentures	0.001
Able to carry out treatment procedures on your own without supervision from your supervisor	0.018

## Data Availability

Data can be accessed from the corresponding author on a reasonable request.
